# Person-Centered Leadership: The Practical Idea as a Dynamic Principle for Ethical Leadership

**DOI:** 10.3389/fpsyg.2021.708849

**Published:** 2021-07-30

**Authors:** Ricardo Murcio, Germán Scalzo

**Affiliations:** ^1^Organizational Behavior Department, IPADE Business School, Mexico City, Mexico; ^2^School of Business, Universidad Panamericana, Mexico City, Mexico

**Keywords:** Carlos Llano, practical wisdom, practical idea, personalism, management

## Abstract

In search of ethical conceptual frameworks that are applicable to the practical reality of companies, ethical leadership has recently gained ground in Business Ethics scholarship as a broad umbrella under which to fit both normative and descriptive approaches to management. This article delves into Carlos Llano's seminal studies in the field, and his rediscovery of the “practical idea” as a dynamic principle for integrating the practice of management and ethical leadership in light of a realistic personalism. Llano was one of the first authors to study the firm from a humanistic, people-centered perspective as a “community or people,” and his view of practical wisdom is an effort to integrate this intellectual virtue with human will by offering a personalist open dynamism that is at the center of all relationships at work, allowing those involved to grow therein. Hence, his notion of the practical idea is his most original contribution to the promotion of managerial action as a catalyst for person-centered leadership.

## Introduction

In recent years, the search for ethical conceptual frameworks applicable to the practical reality of companies has intensified. Accordingly, leadership studies have moved away from transformational toward more ethical perspectives that emphasize the integrity of leaders as both moral managers and moral persons (Treviño et al., 2000), as well as the leader-follower interaction (Avolio et al., 2009). Ethical leadership (Stone et al., 2004; Brown and Treviño, 2006) adds an ethical component to transformational leadership (Graham, 1991), which, in turn, inspires a people-centered management culture, where the interaction between a leader and her followers is a key element for developing the moral dimension of leadership since ethical leaders not only “*do the right thing …* [but also] *show concern for people* through their actions” (Treviño et al., 2000, p. 132, emphasis added).

By exploring the ethical dimension of leadership that displays a true concern for people, this paper delves into managerial (directive) action in light of the “practical idea,” a personalist principle capable of integrating the practice of ethical leadership within a person-centered management culture. As a directive principle (*principium direns*), the practical idea harkens back to the Aristotelian notion of exemplary cause—which Carlos Llano recovered in his effort to develop a proposal for person-centered leadership (Murcio, 2020). It does so by aligning with the broad umbrella of Personalism, “a heterogeneous school of thought that puts the person at the center of all social, political, economic and environmental activity” (Melé, 2009, p. 229).

Indeed, although still nascent and inclusive of a great diversity of approaches (Burgos, 2012), all personalist currents are convinced of the value and dignity of the human person, including tenants like each person is unique and unrepeatable and can never be used as a medium alone; happiness belongs to the personal structure; participation is a characteristic trait of the person in action, analysis of which reveals true transcendence (Melé, 2020, p. 73). In other words, although varied, all personalism coincides in the search for a framework that places the person at the center of the company and considers her its end. For these conceptual frameworks to be viable, the true practice of leadership should be understood as a function of service and collaboration around the common good.

This was precisely the purpose of the late Mexican-Spanish thinker Carlos Llano Cifuentes, who devoted his academic career to the study of the firm, particularly focusing on managerial action in light of the Aristotelian-Thomistic tradition. He continually employed principles related to current theoretical philosophy in an attempt to bring these approaches closer to the practical realm of business (de la Vega, 2009; Jiménez Torres, 2017).^1^ The classical approach to practical wisdom enriched with Personalism and applied to managerial action reveals the possibility of generating a connection between leaders and their teams in line with current efforts to expand the notion of practical wisdom (Akrivou and Scalzo, 2020). Therefore, this article delves into Llano's work in order to trace the personalistic elements that inspired his proposal concerning the relationship between managerial action and practical reason in search of a way to practically integrate all of these features. To do so, we proceed as follows: we start by introducing our author's intellectual program and presenting his characterization of managerial action as a synthesis function, in an attempt to overcome the limitations of the mechanistic approaches that were common in his times. In line with the two managerial functions he identifies—including decision-making and directing people—we proceed to analyze how to properly perform those actions in light of the human being's highest faculties, namely reason in its practical use, and the will. Finally, to build a bridge between theory and practice, we focus on the practical idea, as a dynamic principle that integrates both the ethical side of managerial action and the people-centered focus of a successful directive action into an enriched ethical leadership proposal called person-centered leadership. This paper's ultimate aim is to highlight the central role of the practical idea, which provides space for the creative participation of all individuals in the company, and leads to person-centered ethical leadership.

## Carlos Llano and Ethical Management

The need to establish a dialogue between ethics and management practice is increasingly evident (Moore, 2008; Bachmann et al., 2018) and has inspired in-depth studies of virtue ethics as applied to the company (Solomon, 1992; Ferrero and Sison, 2014; Moore, 2017), and of the central role that practical wisdom occupies in it (Malan and Kriger, 1998; Moberg, 2007; Shotter and Tsoukas, 2014; Scalzo and Fariñas, 2018). This, in turn, has inspired a research field in ethical leadership (Brown and Treviño, 2006) that goes beyond leadership's normative aspect to include other approaches that shift the focus to descriptive features, such as leaders' psychological traits and decision-making, as well as the impact of organizational, cultural or contextual dimensions (Brown and Treviño, 2006). Resulting proposals, which attempt to express leadership's moral dimension, include, among others transformational, virtuous, spiritual or authentic leadership.

In recent years, mainly based on the work of social scientists, focus on leaders' personal dimensions has again shifted to focus on relationships with their followers and the impact on organizational effectiveness (Stone et al., 2004; Correa Meneses et al., 2018). Among these proposals, servant leadership (Greenleaf, 1977; Stone et al., 2004; Spears, 2010) stands out as an approach that is genuinely concerned with serving followers by focusing on their needs and creating opportunities to help them grow within the organization (Luthans and Avolio, 2003). However, it also presents some weaknesses, such as a tendency toward idealism or the risk of manipulation on the part of certain followers who may misunderstand the proper meaning of service (Whetstone, 2002). Indeed, by radically shifting the focus from the leader to the follower, it moves away from directive action's main purpose and, in a certain way, becomes unrealistic and dependent on too many assumptions for its implementation.

A leadership style built around managerial action itself could overcome these limitations by offering a more realistic and balanced approach to directive action from a humanistic perspective, thus building a bridge between theory and practice within the framework of a more robust anthropology that “humanizes the firm” (Llano, 1979, p. 12) without deviating from its main ends. Llano was one of the first authors to study the firm in light of the Aristotelian-Thomistic tradition; his notion of the practical idea is his most original contribution to overcoming the limitations of the so-called classical approach to practical wisdom, in light of the developments of modern and contemporary philosophy, including Personalism. Indeed, his view of practical wisdom is an effort to integrate this intellectual virtue with human will, and to offer an open dynamism that—according to him—Aristotelian and Thomistic thinkers have neglected.

Through the practical idea, personalist leadership that respects the dignity of the person is made possible. This includes, in particular, respecting freedom, which unifies and gives purpose to the tasks and functions proper to organizational life. In other words, it is a practical exercise that opens up the possibility of participation and collaboration for all stakeholders, both when it comes to decision-making and execution. Organizational leaders are responsible for creating working environments in accordance with human nature, making it possible for the people there to live with dignity and in accordance with the personalistic principle that, “No human being should ever be treated as mere means to an end. On the contrary, persons should be treated with respect and also with benevolence and care” (Melé, 2009, p. 232).

Llano recognizes the social nature of the person and, like other personalist authors (Argandoña, 1998; Fontrodona and Sison, 2006; Melé, 2009), understands the company as a community of people whose purpose is found in the common good and development of each of its members, rather than exclusively in the generation of added economic value. Ultimately, companies must serve people so that everyone involved reaches their own end, which must be structured according to happiness and personal ends.

The nucleus of his thought is found in his seminal book *Análisis de la Acción Directiva* [Analysis of Managerial Action] that was originally published in 1979 and is now going on its 15th edition.^2^ In this work, he presents his fundamental interest as well as his intellectual program, which he later deepened philosophically, especially in his books *Sobre la idea práctica* (Llano, 2007) [On the Practical Idea] and *Examen filosófico del acto de la decisión* (Llano, 1998) [A Philosophical Examination of Decision-making], which synthesizes his theoretical proposal on the practical problem: “Many have unsuccessfully tried to find in science... or pure theory... the solution to problems that depend much more on our nerve, temper, character, domain and self-command than on intellect” (Llano, 2007, p. 11).^3^

Llano decided to delve into the notion of leadership after many frustrated attempts to study it in the abundant literature on management. He wanted to find a definition of managerial work that goes beyond its most superficial aspect, but found classic attempts to explain the essence of leadership from scholars like Barnard (1973), Fayol (1949), Mintzberg (1973) and Drucker (2011) as inscrutable and limited to descriptive lists of the functions that a manager undertakes (Llano, 1990, p. 8). In order to offer a definition of managerial action that links the practice of management to practical reason, he instead delved into the philosophical study of human action starting from the Aristotelian-Thomist tradition, anticipating the development that virtue ethics in business has seen of late (Ferrero and Sison, 2014). Llano rejected the notion of a bureaucratic manager merely capable of solving technical problems, as well the emotive version thereof, that of a “stock character” (MacIntyre, 2007, p. 27). He instead advanced the figure of the leader by highlighting the manager as responsible for making the decisions that direct said company toward the common good, promoting a viable scenario of character development for all organizational members (Llano, 2010, pp. 322–325; Beabout, 2012; Moore, 2017).

For Llano, human work must be recognized for its contribution to human flourishing, rather than just for its external results. Thus, he considers management a “domain-relative” that has its own “standards of excellence,” and aims toward improvement of the manager's character (Beabout, 2013). For its part, the company must seek to be more flexible and move away from a mechanistic, scientific conception that aims to structure the organization's processes and policies in such a way that it blocks personal development. For this to be possible, the government of people, rather than of systems, must be considered more important and embodied in the figure of the manager.

To humanize the organization, understanding managerial action in Llano is an important starting point. This process of humanization falls to the manager, who must use practical intelligence in this effort, to effect attitude changes throughout the organization. In order to put this into practice, managerial work rightly understood must become a focal point in business schools and companies, including research that aims to directly impact the world of companies through character education for managers.

## Managerial Action as a Synthesis Function

Llano addresses the issue of managerial action in a specific context, namely the post-industrial world in the second half of the twentieth century, which was characterized by incipient speculation on the phenomenon of the firm from a largely positivist and technical perspective. As one of the first thinkers to philosophically approach the study of the firm as a “community of people” (Llano, 1998, p. 41; Melé, 2012), Llano used a methodology of greater scope and within the framework of a realistic anthropology that gives contemporary man a clearer idea of his work and how to perfect it in organizations. “For Llano, philosophy is a human tendency and a disposition toward the truth... a tendency toward radical, synthetic and plenary knowledge” (Jiménez Torres, 2017, p. 39). Philosophical knowledge is a kind of radical knowledge because it affects and transforms man; it is synthetic because it seeks to overcome the fragmentation of specialization and it is plenary because it aspires to totality or universality (Llano, 2001, pp. 2–4). These factors give Llano's work a truly humanistic character and are especially present in his study of managerial action.

There are many descriptions of what a manager does, he notes, but none of them contains a *propter quid* definition; in other words, it is difficult to identify the reasons for which a given task is managerial and not operational. Llano (1979) studies the firm analytically through the figure of the manager and her action, and generally defines managerial work as “action that does not follow fixed rules and whose results are uncertain” (Llano, 1990, p. 9), although it must be done—he adds—with the aspiration and even obligation of acting correctly, unlike operative work, which follows fixed rules and whose results are, following said rules, at least statistically guaranteed.

Llano identifies two managerial functions, namely (1) decision-making and (2) directing people (Llano, 1979, p. 43), as did (Drucker, 2011, p. 7) and (Barnard, 1973, p. 231). With the former function, he refers to the activity of people who hierarchically make up relevant governing bodies responsible for formulating and defining an organization's objectives and purpose. Decisions concerning the firm's specific objectives are limited by the general purpose of the firm as a social entity. Llano avoids falling into the struggle between generating economic gain or serving society because “in a well-established mercantile society, profit and service are inseparable” (Aspe, 2005). This notion of a company capable of connecting common and individual goods is in tune with the personalism that John Paul II promoted (Melé, 2020).

Thus, the first function of the manager is to guide the organization toward its generic goal, defining the specific actions in which the service it offers is framed and how it generates profits through this end. Once the firm's purpose is defined, the second function is leadership, i.e., directing one's own behavior or that of others toward the stated objective (Llano, 1979, p. 43). Thus, managerial action goes beyond functions performed by a manager.

Llano points out two possible dangers in this definition: (1) that this type of action can be assigned to many activities within the firm since making decisions and directing people is not exclusive to a company's general manager (Llano, 1979, p. 44), but rather happens at all levels of the organization (1998, p. 21) and (2) believing that a manager is made through the position she occupies rather than through her function (p. 19). He concludes by saying, “the manager's function is one of synthesis and interrelation” (p. 21). The interrelational function is of great importance in the definition of “managerial action” since management success depends on the ability to synthesize the firm's apparently divergent aspects. In short, it is “the function of the manager, who attempts to coordinate, rather than just juxtapose, different points of view, see what staff are missing and how, ultimately, with one perspective, another can be improved: This is the work of synthesis, which is a unitary vision absent in the mosaic of the firm. Since a global vision is a must, the manager must have the ability to synthesize” (Polo and Llano, 1997, p. 49). This capacity is revealed in the five aspects Llano considers most relevant (Llano, 1979, p. 44-57), as follows:

1) Synthesis in the firm's functional dimensions. Conceptually, functional areas are “dimensions” or synergistic forces that must be harmonized. A manager is not a specialist, nor should he know or be able to do everything; his function is found in merging the interests of all involved toward the same objective.
2) Synthesis in the seemingly divergent aspects of the firm's overall objective. Each firm's specific reason for being is defined according to its purposes, namely (a) to provide a useful and good service to the community, (b) to generate sufficient economic value, (c) to generate “humane” compensation that allows workers to develop while working and (d) to achieve self-continuity and profitability in the long term. A manager must be able to harmonize two overall objectives, namely service and profit.
3) Synthesis in the firm's structural elements that are seemingly in conflict. Although structural elements—investors, managers and operators—have different interests, they also have their own function and must be compatible for the firm to succeed. Managers must guide these three elements toward the same goal.
4) Synthesis between managerial and operational work. For this, the firm should implement a flexible structure that does not radically separate execution from design, but rather allows all collaborators to exercise managerial and operational functions at different levels, taking into account that a pure function does not exist. Managers must achieve an adequate synthesis to the extent that one is needed.
5) Synthesis between formal and de facto authority. This is based on the distinction between authority—socially recognized knowledge—and power, which is based on a position's power to impose decisions. Managers must maintain both of these elements in their function—with as much authority as possible and as much power as necessary.

## The Role of (Practical) Reason in Managerial Action

Managerial action supposes a type of reasoning to fulfill both its specific objectives and personal character formation, which implies the improvement of intellectual faculties and the development of moral virtues, among which prudence stands out. That is to say, among Aristotle's different uses of understanding and their corresponding perfections (namely, there are three habits of theoretical reason: that of science, that of the first principles and that of wisdom; and two that correspond to practical reason: that of prudence and that of art (Aristotle, 1995, *Nicomachean Ethics* 1139b, [henceforth, NE]), Llano places managerial action among practical reason when he notes that prudence is the habit proper to it (1979, p. 99).

Knowledge is characterized by an attempt to approach the truth; Llano's philosophy begins precisely with a reflection on the notion of truth, which, strictly speaking, can only be speculative, whether it refers to the natural order contemplated by reason or to the order of one's own reason. Llano does not reject the Aristotelian notion of practical truth outright, but he insists that it is better to speak of how truth appears in the acts of the will, and therefore introduces—in light of Aquinas's thought—the notion of the “practical idea”, which he considers more accurate (Llano, 2007). “For Carlos Llano, there is no ‘practical truth’ for a basic, defining issue: truth is always speculative because we consider actions good or bad, right or wrong, rather than true or false, which is a property of judgment and occurs only at the intellective level” (Jiménez Torres, 2017, p. 58).

As we will see, the notion of “practical idea” is at the core of all Llano's thought, which was developed throughout his extensive work and ultimately unveiled the nature of “the practical” in the face of the formation of “practical man,” whose excellence—as seen—is found in the virtue of prudence.^4^ The primacy of practical reason is key in classical philosophy, which is where Llano was most at home philosophically. Therefore, Llano began by methodologically delineating his object of study from a certain metaphysical and anthropological perspective, rather than just epistemologically.^5^

On this basis, he undertook his study of practical rationality, beginning by differentiating—following Aquinas— between speculative and practical intellect, which is the foundation of the distinction between theory and practice (*Summa Theologiae*, II–II, q. 55, [henceforth, STh.]) ^6^. In effect, understanding has two uses, namely theoretical and practical (NE, 1139a) for which human reason—being the same potential—is theoretical or practical according to its use or end (S.Th, I ps., q.79, q. 11). Aristotle calls the former scientific and the latter calculative or deliberative. Although they involve two different ways of knowing, both are included in the same potential, i.e., intelligence, and receive their object from reality. Llano distinguishes these two uses of intelligence by appealing to two underlying reasons: (1) A subjective end: “the practical mind differs from the speculative or theoretical mind in the end it pursues” (Llano, 1979, p. 95), the end of theoretical reason is the knowledge of reality, while practical reason is ordered at the end of the operation (STh, I, q.14–16), that is, its end is human action that it is not universal, but rather contingent (NE, 1095a); and, (2) The ability to bring thought into reality.

Theoretical reason does not change reality when it comprehends, but rather seeks the acquisition of causal knowledge; practical reason, for its part, seeks to transform reality and ends either in the modification of an object (*poiesis*) or of a subject (*praxis*) (Llano, 1979, p. 71–72). Both of these uses of understanding comprehend the objects of reality and aim at truth, but, in the case of theoretical reason, understanding “is simply intellective (that is, speculative) and not practical because it is locked in the mental sphere, in the sphere of pure thought.

One could understand theoretical reason as mental tasks confined to the field of intelligibility and abstraction, and practical reason as capturing the good in a particular object, which, when presented to the will, becomes desirable. According to Sellés (1999), when it comes to practical reason, the object is contemplated in a particular way and the notion of good is added to what theoretical reason contemplates; that is why, for Thomas Aquinas, practical reason is second with respect to theoretical reason “because otherwise we would not know when we are before a *real good* or before an *apparent good*” (Sellés, 1999, p. 27). Thus, theoretical reason precedes practical reason because the latter is capable of directing action (Aquinas, *III Sententiarum*, d.34, q.1, a.2, co; NE, 1138b-1139a).

Theoretical reason distinguishes true goods from apparent ones (Aquinas, *De Veritate*, 21), but just in terms of their truth or falsity and not in light of morality, since it is neither practical nor productive (NE, 1139a). In this sense, “the general and permanent truths belong” to the scientific field (Llano, 2005, p. 22), while practical reason “refers to everything that varies, that is, that can be otherwise...contingent, random or variable” (Llano, 2005, p. 21). Practical reasoning thus deals with the entire spectrum of changing reality, which includes a large part of human actions and experiences and, more specifically, interpersonal relationships (Akrivou and Scalzo, 2020). The bivalent nature of reason is evident; it is an intellectual virtue that perfects reason in its practical function, but it acquires moral character because the first principle of practical reason is based on the notion of good (STh, I-II, q. 94) and moral virtues cannot be exercised without it (Scalzo and Alford, 2016).^7^

## The Role of the Will in Managerial Action

Even though it is possible to differentiate speculatively between theory and practice, “understanding has no other capacity than to penetrate cognitively into reality and to judge it rather than transform it. This conception of understanding as mere and strict reproduction of reality is at the root of all practical action studies” (Llano, 1979, p. 96). Clearly, human action demands a kind of dynamism that theory cannot offer and that is proper to practice itself, with the difficulty that—precisely because it is practical—it cannot be approached from the intellective faculty *a priori* because “there is no theory from which action is born by itself. Speculative theory and practical theory are only distinguished in the concrete” (Llano, 1979, p. 95).

In effect, “not everything that is thought speculatively can be practically realized… because speculative thought is limited to putting concepts in a position to be thought; while practical reason must present concepts that are doable” (Llano, 2007, pp. 144–145). Thus, practical reason appears when action is conceptualized from a position of the doable in the “here and now” of each circumstance, with the particularities of each person, such that “studying human beings with a merely intellective lens falls short if one does not study the motor of actions associated with understanding, which is the will itself” (Jiménez Torres, 2017, p. 54).

Llano then focuses on another, higher human faculty, namely the will, on which focus has been rather residual in the history of thought, in which intellectualist positions have predominated in the ancient world and rationalist ones in the modern world. Thus, “the will is the axis on which Llano's work seems to turn and which unites the speculative with the practical precisely because the will is what makes the theoretical practical… for unraveling the relationships between the speculative and the practical intellect” (Jiménez Torres, 2017, p. 66).

Practical reason is understood thanks to the link between intelligence and will, “and this in a passive and in an active way. It is passive in that it presents to the will the opportunity for action and the objective to be achieved in order for the will to decide, and active insofar as, once a decision is triggered, it directs (‘commands’) the will to execute said decision in a certain way” (Llano, 1979, p. 99). The active mode is properly considered practical; “the passage from the speculative to the practical does not occur with just any incidence of the will. Volitional incidence is undoubtedly required, but not for later bestowing practicality to what was previously thought speculatively. Rather, it is needed for thinking practically from its origin, or for rethinking it and remaking its practical coordinates” (Llano, 2007, p. 41).

In short, Llano intends to argue that, “the practical is already thought of in view of being carried out” (Jiménez Torres, 2017, p. 55), and it is practical precisely because of the will's action that moves understanding to do what one has deliberated in the concrete, the “here and now,” rather than in the abstract. As a faculty, the will moves itself (Llano, 1998, 2005). Here lies the true core of the question because the key to practical reason lies in the *decision of the will* and not in thought (Llano, 2007, p. 80).

Understanding, “when it involves an opportunity for action, has potential to be practical, that is, it can serve practical action. Strictly speaking, we would say that it is speculative knowledge with a transcendental—ontological—relationship to the practical” (Llano, 1979, p. 99). Llano points out that practical reason is “speculative understanding that involves the action of the will” (1979, p. 98) because “a being of action depends on the will as its efficient cause; but the specific determination of action comes from the understanding that directs the will in the manner of its formal cause” (p. 99). After that understanding judges a given opportunity and deliberates to set an objective, the will decides to carry out the given task. Once a decision is made and “while it subsists dynamically, understanding directs the execution of the action, which means that understanding is practical not just as a receiver of opportunities (passive practicality), but also as director of the execution of action that the will has already decided upon” (Llano, 1979, p. 99-100). The *essence* of action depends on the will as its efficient cause (Llano, 1979, p. 13), and on understanding as a formal cause.

For Llano, practical knowledge is characterized by the intervention of the will, which chooses a good captured by practical, rather than theoretical, reason. The result of what theoretical reason grasps is pure speculation; it is knowledge of reality and, therefore, passive abstraction. On the other hand, the technical conception of the firm assumes that scientific, speculative knowledge is best for management.

## Integrating Practical Reason and the Will in Managerial Action

For Llano, the cycle of managerial action is directly related to the acts of practical reason. There are three activities typical of managerial action, including diagnosis, decision and command. It is a common mistake to reduce practical rationality to its speculative aspect, which Llano calls diagnostic and defines as “knowledge by which we capture opportunities for action, as well as build our ability and resources to take advantage of them, from the contingent, fleeting and particular facts of an event” (Llano, 1979, p. 155). While the former (diagnosis) is closer to the speculative field, the others relate more to the practical field (Llano, 1979). These three moments are related in the order of practical intellect according to Thomas Aquinas (Jiménez Torres, 2017, p. 56; Llano, 1979, p. 100). To better understand this relationship, we will delve into Aquinas's study of practical reason's acts and habits.

(1) The first approach to reality is the practical apprehension in its two modalities, namely theoretical and practical. “Practical reason also apprehends reality, but to the apprehension of reality as truth, proper to theoretical reason, the practical apprehension adds that of the good” (Sellés, 1999, p. 44). Thus, the first of the acts is simple practical apprehension, which occurs when contemplating the object as good or possible. In this double apprehension of truth and the good, we can see that practical reason is second with respect to theoretical reason because, otherwise, a true good could not be distinguished from an apparent good. This act of practical reason is followed by its counterpart in the will, which is the acceptance of the good: saying yes to that which practical reason proposes as good. The will does not simply accept what is presented by practical reason because the will is interested in having many possibilities among which it can decide since none of them is necessary, which is why acceptance is required to continue with the act.
(2) Once the end is grasped and accepted, we proceed to the second act of practical reason: council (*consilium*), which is an inquiry of practical reason about the most appropriate means for achieving an end (García López, 2006, p. 154). Council is an immanent act that deals with mediated goods rather than those that deliberate on the end. It refers to the act of thinking rightly for oneself— or with the help of others—the possible means for achieving an end. This exercise culminates in a perfection of reason called *eubulia* (Sellés, 1999, p. 45). The latter act of practical reason is followed in the will by its counterpart, which is consent. The act of consent (*cum-sentire*) is carried out in the will where it adheres to that which has been determined; it permits different means without deciding for any, but rather validates the different options.
(3) Deliberation is followed by practical judgment (*iudicium practicum)*, which is to recognize, among all the appropriate means, a determined path toward the end as the most appropriate to follow (Pieper, 1974, p. 59). This act occurs with a significant mix of reason and will; the former involves highlighting and comparing options to present to the will, from which choice follows. Here, Aquinas explains in *Q. D. D. Veritate*, q.22 (Sellés, 1999) that it is an act of the will for the reason of its object, the good, and by reason of its very act, because choice is the last step in acceptance. This act refers to the will highlighting one possibility in particular among the ones presented in the council such that it is “an act of the will that presupposes nevertheless an act of understanding” (García López, 2006, p. 154). The choice for something concrete excludes all other possibilities, which remain at the level of options that could have been chosen, but that were not. The habit that perfects practical reason in the order of practical judgment (Gorce, 1928) is called *synesis*, meaning determining well. *Synesis* is only judicious and thus is different and previous to command; it is still in the order of means because, although it calls attention, it does not carry anything out.
(4) The last act is command (*imperium*)– also called precept (*praeceptum)*–, which tells the will to act and whose habit is the most important among practical reason, namely prudence. Prudence “it is right reason about what can be done” (NE, 1140b) and its purpose is to determine what should be done or not. The first acts remain at the level of thought or understanding with a view toward action, for which it is indispensable, but definitively prior. Prudence commands and orders execution; its counterpart in the will is practical use by which it governs the moral virtues in undertaking decisions. For this reason, it is said that prudence commands. It enhances the virtues and their ability to command or govern others.

**Figure 1:**
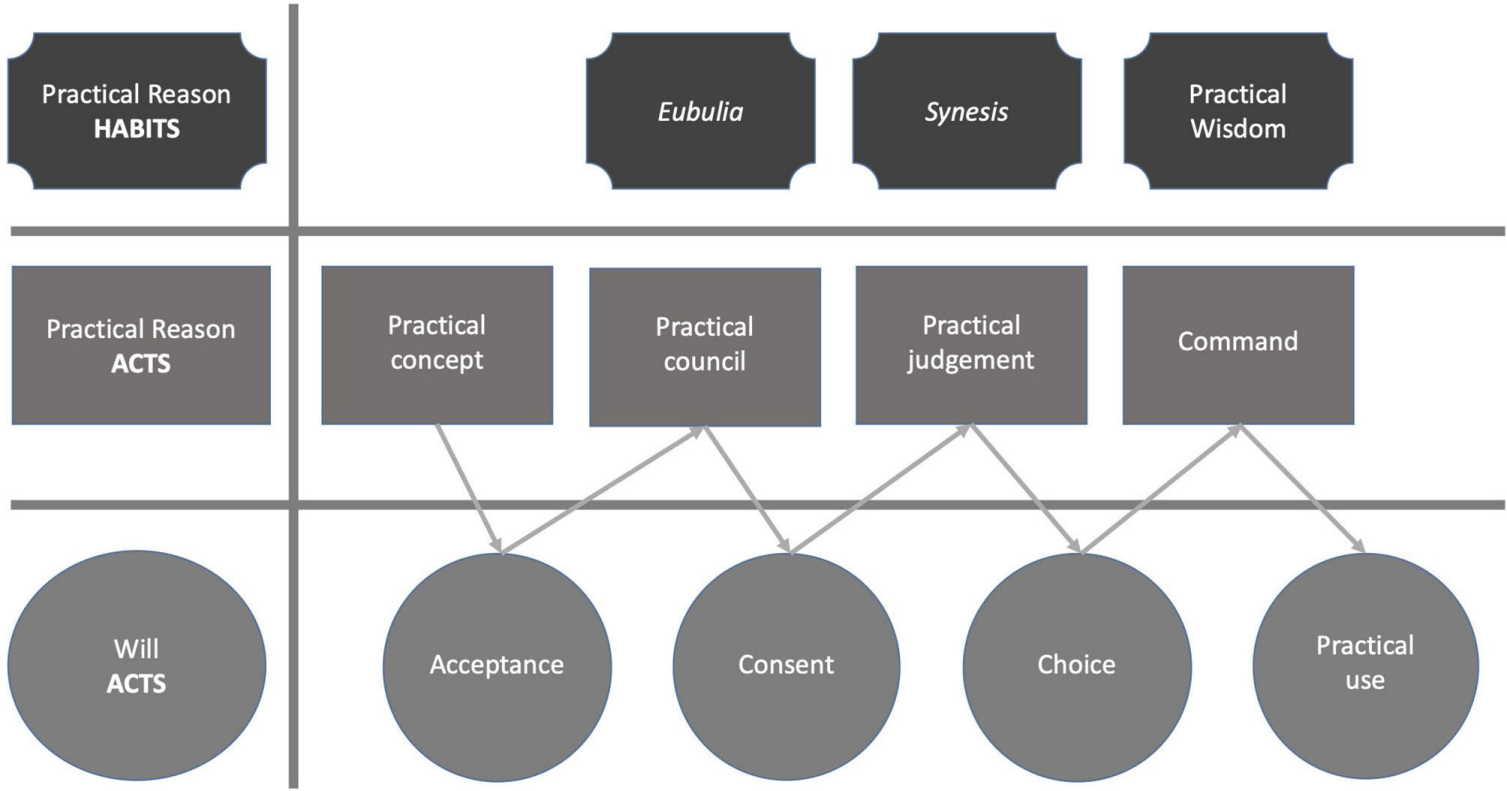
Practical reason and will: acts and habits.

By way of synthesis, in addition to simple practical apprehension, practical reason has three other acts: (1) the practical council, (2) the practical judgment and (3) command (STh, II-II ps., Q. 153, a. 5, co.). Each has a habit: (1) *eubulia*, (2) *synesis* and (3) prudence, respectively. Likewise, each act of practical reason corresponds to an act of the will: (1) simple practical apprehension is followed by acceptance, (2) council is followed by consent, (3) practical judgment is followed by choice and (4) command is followed by practical use. The Figure 1 summarizes this scheme.

## From Management to Leadership: the Practical Idea

As mentioned, managerial action is not based on scientific principles, but rather on practical and contingent ones because “the ability to think well does not coincide with the ability to decide well... ideal solutions do not exist for real problems, nor do ideal objectives exist for real opportunities” (Llano, 1979, p. 71, 2005). Moreover, decisions must consider that the materialization of ideas depends on many circumstantial and contingent factors, as well as the character of the decision maker herself (Llano, 2005). Thus, in practical life, we should look for the right decision rather than the truth (Llano, 2005, p. 22). The possibility of getting it right when dealing with these situations implies being able to effectively carry out a decision. Leadership is therefore possible thanks to the ability to be right in practice; “to be prudent is to hit the nail on the head or, as Aristotle says, it is possible to err in many ways, but you can only be right in one way” (Sellés, 1999, p. 32).

The core notion of Llano's philosophy lies precisely at the intersection between the theoretical and practical fields; the practical idea, which he considers a “pretext” for illustrating the paradoxical task of characterizing practice starting from theory and giving the will the place it deserves in anthropology (Llano, 2007, p. 11). This notion is Llano's most original contribution and involves an implicit leap from the plane of essences to that of real existence or, we could say, from metaphysics to anthropology.

The mission of the idea, which is born in understanding and becomes practical through the exercise of the will, is to change extra-mental reality. Llano is radical on this matter as he argues that “either an idea is practical or it is not an idea” (2007, p. 12). As seen, the speculative intellect is distinguished from the practical intellect by subjective purpose and results; in other words, theory ceases to be so when a person proposes an end and achieves it. For Llano, knowledge considered in itself is mere speculation, but “whenever it involves an opportunity for action, it has the potential to be practical” (1979, p. 99). Theoretical reason knows reality; practical reason discovers the opportunities to transform it. The agent chooses to perform one of them through the influence of the will: “Here, insofar as understanding directs practical execution, it can absolutely be deemed practical” (Llano, 1979, p. 99).

Managerial action is a practical knowledge and, as such, must correctly carry out what it has decided. Practical knowledge takes precedence over strictly theoretical knowledge in managerial functions. This leads to the difficulty that practical truth cannot be known through science or axioms, but that it is a kind of knowledge that is perfected through experience and the exercise of prudence. There is an inseparable link between experience, perseverance and practical reason, and precisely for this reason it is important to study how human beings discover the exemplary cause or practical idea. In order to do so, Llano carries out an unusual and totally novel study that is ultimately a fundamental contribution to managerial action and classical philosophy.

As (Jiménez Torres, 2017, p. 66) illustrates, Llano often quoted Chesterton to exemplify the aforementioned, differentiating between two classes of idealists: those who idealize reality and those who realize the ideal, with the latter included in his corpus under the notion of practical idea. The former stance is in line with Platonic thought, while the latter is located in the Aristotelian tradition that sees ideas as useless for explaining the origin of movement, that is, of practical action. The idea must be practical to bring it to fruition.

To explain practical action without compromising freedom (Llano, 1983, 2005) Llano focuses on the importance of postulating the existence of a guiding cause, which is precisely what Aquinas does with his notion of directing principle—*principium dirigens* (Llano, 2007, p. 86). This corresponds with the Aristotelian notion of exemplary idea: “something thought by the architect with the intention of doing it in practice” (Llano, 2007, p. 19), and that is decisive for conceiving of the idea as an exemplary cause.^8^ Thus, “[t]he exemplary cause is only the idea that has an efficient cause endowed with intelligence” (Llano, 2007, p. 20). We must not confuse “exemplary idea” with “exemplary cause” because an idea may not materialize in reality (it may stay in a state of potential or as an idea alone, that is, as “understood form”), but exemplary cause—precisely because it is a cause—must act on its causation (Llano, 2007, p. 141). This implies the intervention of the will because “in the exercise of the act, the will exercises its superiority over understanding, and understanding has to obey its mandates” (Jiménez Torres, 2017, p. 67). Thus, an exemplary cause is synonymous with a practical idea, but not with an exemplary idea (Llano, 2007, p. 86). The notion of practical idea is one of the most difficult concepts in Llano's work because it is ambiguous in that it is speculative and practical at the same time (Jiménez Torres, 2017, p. 63).^9^

In Llano's corpus, the exemplary idea also expresses an understanding of man as an open and free system (Polo and Llano, 1997),^10^ which is an approximation to the condition of being a person. The exemplary idea is, at the same time, a speculative, “polyvalently causal”^11^ notion (Llano, 2007, p. 82) and “an open,” and even inclusively practical, “regulatory process” “because not only is it useful for changing external things, but also for what is most fundamental, namely changing one's self according to a model of life, a style of existence, a pattern of being that I must discover, accept and conserve” (2007, p. 14).

In this way, Llano's main contribution to this topic emerges, namely that the key element to practical life is not prudence or practical wisdom—an intellectual habit—but rather is the practical idea—a regulatory and open process. As Zagal points out, the exemplary cause as an idea of an intelligent agent is not enough for Llano; for him, “the exemplary cause has to incorporate certain dynamism, it must be susceptible to changing on the fly. Thus, an authentically practical idea is not set in stone and is not a conceptual fossil, but rather is an outline open to feedback” (Zagal, 2005, p. 348). Thus, the practical idea contains the prospect of the meaning of human life insofar as “the agent's project is gradually perfected as it is set into motion” (Zagal, 2005, p. 349). This feedback as an expression of dynamism, which, according to Llano, Aristotelianism and Thomism neglected, is an attempt to get closer to the truth about man because man's truth is characterized by its dynamic integrity (Polo, 2003).

For Llano, the practical idea, an open and dynamic process, helps understand collaboration in the execution of tasks, thus offering all who collaborate in an organization the opportunity to cease being “mere” means or “resources.” Herein, employees are treated in a way that corresponds to their dignity as persons. Leadership, as mentioned, is a practical activity, rather than a scientific exercise, because it is not speculative.

Leadership that respects the dignity of the person must be able to unite organizational objectives with those of the individuals who work there. Knowledge of the person, as noted, is essential for achieving this, and involves dialoguing with one's team in communication and especially in participation. The latter, for Llano, is understood as a “common doing,” sharing the company's vision with the aim of integrating the individual into it. Communication goes “beyond the simple informative nature of mandates since they derive from motives and ends that must be known and shared with those who are to obey them.” (Polo and Llano, 1997, p. 130). They can be shared thanks to the dynamism of the practical idea since “authority by conviction cannot be separated from a decision-making system by participation” (Polo and Llano, 1997, p. 239). Ultimately, this involves discovering the power of participation for achieving results.

The act of managing– for Llano– involves three moments: (1) fluid communication, which implies participation in seeking the best alternatives for action, (2) good decision-making, as a result of an arduous and open dialogue process, as well as the active involvement of all members and (3) better results, which are the product of execution that arises from real connection to the project of which the collaborator forms part. Collaborative participation in decision-making increases efficiency; therefore, “participating in deliberation helps one understand mandates, which is required for their execution” (Polo and Llano, 1997, p. 131). This is a better system because it motivates participants to understand reasons and motives, making their work their own and encouraging the sharing of results. For this to be possible, participants must be trained and educated in decision-making. Moreover, the practical idea gathers a horizon of meaning for human life, insofar as it corresponds to the agent's life project, which is gradually perfected to the extent that it is set in motion. In this way, it is not just useful for decision-making on external matters, but also for changing one's self according to a model of life that must be discovered in common.

The leadership style that Llano proposes, which requires conviction rather than mandate, can be seen in the ability to influence that it generates. This influence requires the generation of trust not as a managerial strategy, but with the true intention of knowing others and helping them build their own life projects. A leader's reputation is key in the facilitation of this process; ethical leaders must be seen as moral persons (Treviño et al., 2000), as well as role models, to create a space for friendship in which they can work closely with their followers (Brown and Treviño, 2006). As Álvarez de Mon (2000) points out, trust cannot be ordered, only aroused, provoked, encouraged, induced. This is because the most precious part of human relationships are free goods (Álvarez de Mon, 2000, p. 49). The true depth of the human person is only reached through personal interaction and friendship. In a model that establishes the person as an end, authority is characterized by respect for freedom, which is manifested when each person is able to develop their own personally chosen project. The company must support and act as a resource for achieving each individual's ultimate ends. Thanks to the practical idea, diverse stakeholders can contribute to a common good in practice rather than with mere intention.

In practical terms, this has important implications for organizational life since it allows for the merely practical elements of managerial activity to effectively generate growth, both at the personal and organizational levels. One successful case can be found in the example of Grupo Bimbo and its former CEO and co-founder Lorenzo Servitje, who, like many other businessmen, was directly influenced by Llano's ideas thanks to their close friendship at IPADE Business School. Servitje has received countless acknowledgments for his exemplary management, and is considered an exemplary model of ethical leadership, while Grupo Bimbo has become an international benchmark in ethical management, sustainability, and social responsibility.^12^

## Conclusion

This paper introduced Llano's work on managerial action in light of practical wisdom. It made a systematic account of the acts and habits of practical reason in the Aristotelian-Thomistic tradition, as well as focused on the will– whose study has been rather overlooked. Then it presented a synthesis of Llano's most original contribution, namely the *practical idea* or exemplary cause, which is precisely what allows firm managers to go beyond mere speculation toward the practice of leadership.

Thus, Llano's main contribution is found in having formulated the key element for practical life as a practical idea: an open and regulatory process that goes beyond practical wisdom, which is an intellectual habit, to integrate the will in interaction with other people's freedom. Certainly, the perfection of the entire managerial cycle consists in executing its essential acts in a constant manner and according to reason, but the main function of decision-making in Llano's managerial action is to think practical ideas because, without them, managers could not guide their own action or that of others.

Llano's theory of managerial action is indeed fruitful and original. It is worth mentioning two aspects that derive from it: (1) the final end of organizational action is a cause and not a result: the exemplary cause is the end of the decision-making function; starting from a plan is essential for comparing whether the results are correct according to their dynamic relationship with the practical idea; and (2) the practical idea is *principium dirigens* and, as such, it should be able to be carried out in all practical activity and at all levels—both directive and operational. In accordance with Llano's inclusive view of the firm, the exemplary idea is not reserved for managers alone, but rather is open to all who participate in a common project that contributes to their own perfection and development. The firm must be an exemplary place for character education because each of its members direct in some way, although with varying scopes and levels of influence.

We have shown how this personalist proposal constitutes a promising, important and urgent path in the context of work and management. Person-centered leadership can inform ethical leadership in a humanistic way by providing deeper, more profound foundations for engaging in interpersonal relationships that are conducive to mutual growth.

## Author Contributions

RM and GS: conceptualization, formal analysis, methodology, validation, writing—original draft, and writing—review and editing. Both authors contributed equally.

## Conflict of Interest

The authors declare that the research was conducted in the absence of any commercial or financial relationships that could be construed as a potential conflict of interest.

## Publisher's Note

All claims expressed in this article are solely those of the authors and do not necessarily represent those of their affiliated organizations, or those of the publisher, the editors and the reviewers. Any product that may be evaluated in this article, or claim that may be made by its manufacturer, is not guaranteed or endorsed by the publisher.

## Footnotes

^1^Llano relies on this tradition in the *Angelicum*, where he refers to Professor Garrigou Lagrange as his main influence. This starting point connects him directly with the principles of personalism, especially as seen in the writings of John Paul II. Llano was a prolific author (with 42 books and 245 articles) and devoted a good part of his academic career to the study of practical rationality and its relationship to managerial action.

^2^All quotes from Carlos Llano's works have been translated into English by the authors.

^3^His work around this thematic axis is wide and varied. He devoted much attention to issues related to business and organizational culture, ethics and social theory. See Jiménez Torres (2017, p. 31–34) and Murcio (2020).

^4^See Aquinas *STh*, II–II, q. 50. There is a progressive distancing from Aristotelian ideas in late Scholasticism, see for example Gillespie (2008) and Gilson (1955).

^5^Among his writings on speculative philosophy, the tetralogy “Bases noéticas para una metafísica no racionalista” (Noetic Bases for a non-rationalist metaphysics) is especially noteworthy. There, he exhaustively studies the acts of human knowledge, which include *Abstractio, Separatio, Demonstratio and Reflexio*. See Jiménez Torres (2005).

^6^According to Sellés (1999), Aquinas addresses the issue of prudence mostly in *In IV Sententiarum*, d. 33, q. 2 and 3; *Summa Contra Gentiles*, III, 35*; STh*, II-II ps., Qq. 47–56 and II-II ps., Q. 182.

^7^Moral matter has certain characteristics, including the fact that it is particular because it focuses on the concrete; it is also contingent or non-necessary since it can unfold in several ways. It is doable because only humans can realize it and it is temporary like everything that changes.

^8^Technically, the exemplary cause is an extrinsic formal cause, as Aquinas explains in *In Metaphysicam*, V, lect2, n764, following Aristotle in *Metaphysica*, V, 2, 1013th 27 (cited in Llano, 2007: 19; 21). According to Llano, this topic was not given sufficient importance because of the dangerous analogy with the problem of exemplary ideas in divine understanding (Llano, 2007: 20-22).

^9^Llano's study of this concept (2007) is thorough and very well documented, the result of several decades of reflection, as seen in the prologue to the second edition. Some philosophers have entered into dialogue with this proposal, among which Zagal (2005) stands out, but, due to its difficulty and importance, it certainly could use more philosophical attention.

^10^Open refers to the fact that it is exposed and that it is demonstrative. Human beings express themselves from their intimacy. The expression “from intimacy” guarantees the unity of human actions and, therefore, that they are all related and integrated in the same person. In addition, as this opening is never lost, growth is unlimited: the relationships between the elements of an open system are increasingly intense and integrated. This free concept indicates that growth's direction is not fixed and, therefore, both positive and negative growth can occur. Positive growth integrates, meaning it “goes from less to more…[and] requires learning. Learning is not easy because it consists of integrating some novelty into what has already been achieved” (Polo and Llano, 1997, p. 58).

^11^It is polyvalent causal because, according to Llano, the other four causes with which the exemplary cause has a close relationship converge in it intersectionality rather than tangentially (Llano, 2007, p. 14). Llano takes up the study of causes in Aristotle, starting with Book II of Physics, chapter 3 (Bk 194b, a, 20–35). At the end, he notes, “in another sense, the form and the model are called cause” (Llano, 2007, p. 104). Llano indicates that the great majority of philosophers accept tetracausality: material, formal, efficient and final causes, leaving the exemplary cause as an extrinsic cause within the formal one. Instead, he argues that there are five at the same level, since one thing is the form that remains in the object and another the model from which it is made. Llano proposes retrieving it because it is valuable for management. For him, it is clear that there are, at least, two causes: the form and the model, and that both can be identified in the essence. Garrigou-Lagrange has studied this subject through a theological lens.

^12^See: https://www.grupobimbo.com/es/reconocimientos/general, Retrieved 2021-06-23.
